# Stabilized Polymer Micelles for the Development of IT-147, an Epothilone D Drug-Loaded Formulation

**DOI:** 10.1155/2016/8046739

**Published:** 2016-12-01

**Authors:** Adam Carie, Bradford Sullivan, Tyler Ellis, J. Edward Semple, Taylor Buley, Tara Lee Costich, Richard Crouse, Suzanne Bakewell, Kevin Sill

**Affiliations:** Intezyne Technologies, 3720 Spectrum Blvd. Ste. 104, Tampa, FL, USA

## Abstract

Epothilones have demonstrated promising potential for oncology applications but suffer from a narrow therapeutic window. Epothilone D stabilizes microtubules leading to apoptosis, is active against multidrug-resistant cells, and is efficacious in animal tumor models despite lack of stability in rodent plasma. Clinical development was terminated in phase II due to dose limiting toxicities near the efficacious dose. Taken together, this made epothilone D attractive for encapsulation in a stabilized polymer micelle for improved safety and efficacy. We have designed a library of triblock copolymers to develop IT-147, a lead formulation of epothilone D that extends plasma circulation for accumulation in the tumor environment, and potentially decrease systemic exposure to reduce dose limiting toxicities. The drug loading efficiency for IT-147 exceeds 90%, is 75 nm in diameter, and demonstrates pH-dependent release of epothilone D without chemical conjugation or enzymatic activation. Administration of IT-147 at 20 mg/kg increases exposure of epothilone D to the plasma compartment over 6-fold compared to free drug. At the same dose, 20 mg/kg epothilone D from IT-147 is considered the no observed adverse effect level (NOAEL) but is the maximum tolerated dose for free drug. Consequently, IT-147 is positioned to be a safer, more effective means to deliver epothilone D.

## 1. Introduction

Systemic administration of cytotoxic drugs, accompanied by their severe dose limiting toxicities, is standard practice in oncology. The expansive search for new drugs through natural product discovery and rationally designed chemical libraries have identified an abundance of therapeutic agents with new mechanisms of action and potential for difficult-to-treat diseases such as cancer. Epothilones are a class of cytotoxic macrocyclic compounds originally discovered in the mid-1980s isolated from myxobacteria* Sorangium cellulosum* [1–3]. Epothilones A and B were isolated and determined to have taxane-like activity in 1993 [4]. Epothilone B was found to be a potent inhibitor of tubulin polymerization and was shown to displace bound paclitaxel on microtubules [4, 5]. Total synthesis of epothilones A and B was published by 1997, and family members C–F, along with libraries of analogues, were subsequently investigated for antitumor activity [6–11]. The epothilones demonstrated potent in vitro inhibition of cancer cell growth with low nanomolar IC_50_ values and maintained activity against cell lines that demonstrate multidrug resistance, including paclitaxel cross-resistance [8, 12–16]. Epothilone B demonstrated the highest potency of the family of epothilones. In vivo, epothilone B dosed by intraperitoneal bolus in mouse tumor models demonstrated a maximum tolerated dose of 0.3 mg/kg, with an LD_50_ of 0.6 mg/kg, compared to epothilone D which demonstrated a maximum tolerated dose of 25 mg/kg on the same dosing schedule [13]. Although epothilone D was better-tolerated, clinical development was halted after phase II studies demonstrated a poor response rate at the maximum tolerated dose [17–19]. An analogue of epothilone B, ixabepilone, was approved by FDA for treatment of refractory breast cancer, although some clinical studies have indicated that ixabepilone is not superior to paclitaxel in treating refractory solid tumors [20–22]. The epothilones have demonstrated promise as potential anticancer treatments but suffer from a narrow therapeutic window. Therefore, formulations of epothilones that improve tolerability while maintaining efficacy should demonstrate clinical utility.

Many different drug delivery technologies, including liposomes, degradable polyester particles, and polymer micelles, have been investigated both preclinically and clinically, but few have achieved FDA approval [23]. There are many factors to consider when designing chemotherapeutic nanoparticles, including size, shape, payload capacity, targeted drug release, and stability [24]. We have hypothesized that stability of the nanoparticle after administration is the most important aspect in the clinical success of this class of drug delivery technologies. In our investigation of polymer micelle delivery systems, we have designed a library of triblock copolymers to encapsulate hydrophobic chemotherapeutic agents in micelles that are stabilized to dilution in the circulatory system. The amphiphilic block of the polymer is composed of 12 kDa poly(ethylene glycol) (PEG), and the hydrophobic core-forming block is composed of mixture of amino acids, as shown in Figure 2. The stabilizing block incorporated between the PEG block and core block differentiates this approach from the abundance of polymer micelle formulations reported previously. The stabilizing block in the polymers investigated for epothilone encapsulation is based either on iron-hydroxamate bonds formed among hydroxamic acid (NHOH) residues on polymer chains or on disulfide bonds formed between cysteine (Cys) moieties in the polymer chains (Figure 2). The ability to stabilize a drug-encapsulated micelle for systemic administration without the need to chemically conjugate the drug to the polymer allows for extended circulation time in the plasma compartment, time to accumulate in the tumor environment, and the ability to release payload without reliance upon enzymatic activity for cleavage. Stabilizing the drug-loaded polymer micelles by forming iron-hydroxamate dative bonds allows for a pH-dependent release of the dug payload. The iron chelated hydroxamic acid forms a stable bond at physiological pH, which subsequently becomes unstable as the pH decreases. This allows for a triggered release mechanism in the acidified tumor microenvironment, as well as in endosomal/lysosomal compartments upon intracellular transport [25, 26]. Stabilizing polymer micelles with disulfide bonds allow for targeted release of the drug payload upon cellular entry based on the oxidative potential in tumor cells [27]. These release mechanisms are represented graphically in Figure 3. Formation of drug-loaded micelles with these triblock copolymers typically represents a 70–90% efficient process, and results in nanoparticles with average diameters between 60 and 100 nm. Herein we discuss formulation and lead optimization of epothilone D in stabilized triblock copolymer micelles and physiochemical characterization of a small library of formulations.

## 2. Materials and Methods

### 2.1. Materials

Epothilone B was purchased from Toronto Research Chemical (Toronto, Ontario, Canada) and epothilone D was purchased from Austin Chemical (Buffalo Grove, Illinois). Dichloromethane, ferric chloride, phenylmethylsulfonyl fluoride (PMSF), cremaphor, dithiothreitol (DTT), anhydrous dichloromethane (DCM), anhydrous* N,N*-dimethylformamide (DMF),* N*-methylmorpholine (NMM), 4-dimethylaminopyridine (DMAP), and lithium hydroxide monohydrate (LiOH·H_2_O) were purchased from Sigma-Aldrich (St. Louis, Missouri). Trehalose was purchased from Carbosynth (Berkshire, United Kingdom). Dialysis tubing with 3500 molecular weight cut-off (MWC) and tangential flow filtration filters with 10 kDa pore size were purchased from Spectrum Labs (Rancho Dominguez, California). Pass-through sterile 0.22 micron filters were purchased from Sartorious (Goettingen, Germany). Thermo Syncronis C18 HPLC column (97105-254630), HPLC grade methanol, HPLC grade acetonitrile,* n*-butyl chloride, absolute ethanol, K_2_EDTA tubes, black wall, clear bottom 96-well plates, tetrahydrofuran (THF), isopropyl alcohol, methyl* tert*-butyl ether (MTBE), acetic anhydride (Ac_2_O), acetone, and acetic acid were purchased from Fisher Scientific (Waltham, Massachusetts). Absolute ethanol was purchased from Decon Labs. Hydroxylamine (50% aqueous solution) was obtained from Alfa Aesar (Haverhill, Massachusetts).

### 2.2. Triblock Copolymer Synthesis

**Figure 1:**
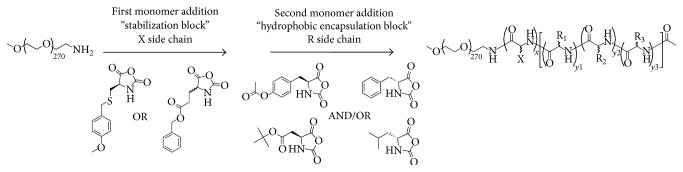
General polymerization scheme for triblock copolymers. Monomer addition by living polymerization followed by sequential functional group manipulations yields triblock copolymers for formulation of drug-encapsulated polymer micelles.

All NHOH or Cys triblock polymers were synthesized from in-house prepared mPEG-NH_2_ and *α*-amino acid* N*-carboxyanhydrides (NCAs) using previously published procedures and as a demonstrated in Figure 1 [28, 29]. As a specific example, the synthesis of ITP-103 mPEG12K-NH-*b-p*-[D-Glu(NHOH)_5_-*co*-L-Glu(NHOH)_5_]-*b-p*-[D-Phe_15_-*co*-L-Tyr_15_-*co*-D-Leu_10_]-Ac is as follows: synthesis of mPEG12K-NH-*b-p*-[D-Glu(OBn)_5_-*co*-L-Glu(OBn)_5_]-*b-p*-[D-Phe_15_-*co*-L-Tyr(OAc)_15_-*co*-D-Leu_10_]-Ac (ITP-103 precursor): mPEG12.7K-NH_2_ was dried via azeotropic vacuum distillation with toluene to remove trace amounts of moisture. The dry PEG was dissolved 0.1 g/mL in a mixture of 2 : 1 v/v anhydrous DCM and DMF. D-Glu(OBn) NCA (5 eq.) and L-Glu(OBn) NCA (5 eq.) were added and the reaction was stirred at room temperature. After ~16 hrs, gel permeation chromatography (GPC) analysis indicated complete consumption of the NCAs. D-Phe NCA (15 eq.), L-Tyr(OAc) NCA (15 eq.), and D-Leu NCA (10 eq.) were added and the reaction was stirred for ~21 hrs, at which point the reaction was complete (GPC analysis). Ac_2_O (10 eq.), NMM (11 eq.), and DMAP (1 eq.) were added and the reaction was stirred overnight before diluting with DCM and filtering through a glass microfibre filter. The reaction mixture was concentrated under reduced pressure to remove the DCM, transferred to a large polypropylene bucket, and precipitated by the addition of isopropyl alcohol (~8–10 volume equivalents) with vigorous mixing. The solids were collected and washed with MTBE (6–8 volume eq.) and then dried* in vacuo* to yield the title compound as a fine cream-colored powder (yield typically > 95%). Synthesis of mPEG12K-NH-*b-p*-[D-Glu(NHOH)_5_-*co*-L-Glu(NHOH)_5_]-*b-p*-[D-Phe_15_-*co*-L-Tyr_15_-*co*-D-Leu_10_]-Ac (ITP-103): mPEG12K-NH-*b-p*-[D-Glu(OBn)_5_-*co*-L-Glu(OBn)_5_]-*b-p*-[D-Phe_15_-*co*-L-Tyr(OAc)_15_-*co*-D-Leu_10_]-Ac (ITP-precursor) was dissolved 0.1 g/mL in anhydrous THF with the assistance of gentle heating. Once cooled to ambient temperature, LiOH·H_2_O (25 eq.) and NH_2_OH (50 wt.% in H_2_O, 125 eq.) were added. The reaction was monitored via ^1^H NMR (400 MHz, DMSO-*d*_6_) for the disappearance of the -CH_2_OBn and -OAc protons and was typically complete after 24–36 hrs. Acetic acid (125 eq.) and acetone (1250 eq.) were added and the reaction mixture was heated until reflux and then left to stir and cool to ambient temperature overnight. The reaction mixture was filtered through a glass microfibre filter and transferred to a large polypropylene bucket. The product was precipitated by the addition of MTBE (~8–10 volume eq.) with vigorous mechanical stirring. The solids were collected, washed with absolute ethanol (~5 volume eq.) and then isopropyl alcohol (~1 volume eq.), and dried* in vacuo* to yield the title compound as a fine light cream-colored granular solid (yield typically > 90%).

### 2.3. General Method for Formulation of Epothilone D Loaded Micelles (Hydroxamate)

Formulation of epothilone-loaded micelles was performed using an oil in water emulsion technique. Polymer was dissolved in water, and epothilone D was dissolved in a solution of 20% methanol in dichloromethane. The feed ratio for epothilone to polymer was 5% (w/w). The organic solution containing epothilone D was added to the aqueous polymer solution while stirring under high shear using a Silverson L4RT rotor/stator mixer with a fine emulsor screen. The resulting emulsions were stirred in a fume hood for 12 hours to allow the evaporation of the organic phase. Ferric chloride solution (10 mM final concentration) was added to form iron-hydroxamate bonds between polymer strands for micelle stabilization for formulations with polymers containing a hydroxamic acid stabilizing block and was then purified and concentrated by tangential flow filtration (TFF). Trehalose (50% w/w polymer) was added as a cryoprotectant, and the solution was filtered through a 0.2 *μ*m filter and lyophilized.

### 2.4. General Method for Formulation of Epothilone D Loaded Micelles (Disulfide)

Formulation of epothilone-loaded micelles was performed using an oil in water emulsion technique. Polymer was dissolved in water containing dithiothreitol (DTT, 20 mM), and epothilone D was dissolved in a solution of 20% methanol in dichloromethane. The feed ratio for epothilone to polymer was 5% (w/w). The organic solution containing epothilone D was added to the aqueous polymer solution while stirring under high shear using a Silverson L4RT rotor/stator mixer with a fine emulsor screen. The resulting emulsions were stirred in a fume hood for 12 hours to allow the evaporation of the organic phase. The solution was then subjected to diafiltration by TFF to remove dithiothreitol for stabilization of the micelles by interstrand disulfide bond formation and then concentrated. Trehalose (50% w/w polymer) was added as a cryoprotectant, and the solution was filtered through a 0.2 *μ*m filter and lyophilized.

### 2.5. Assay for Epothilone D Drug Loading

Drug loading of epothilone micelle formulations was performed by HPLC/UV using an Waters Alliance 2695 HPLC separations module equipped with a 2998 photodiode array detector (Milford, Massachusetts). Separation was performed using a Synchronis C18, 5 *μ*m, 250 × 4.6 mm HPLC column. Mobile phase A consisted of water with 0.2% acetic acid, and mobile phase B consisted of methanol. Flow was isocratic at 1.2 mL/minute and consisted of 15% mobile phase A and 85% mobile phase B. Injection volume was 10 *μ*L. Analyte was monitored at 249 nm with retention time at 7.0 minutes and a total run time of 10 minutes. Formulations were dissolved in methanol at a concentration of 2 mg/mL, and epothilone peak area was compared to epothilone standards in a range of 20–100 *μ*g/mL in methanol. Epothilone concentration in the formulations was then converted to percentage of epothilone content to polymer content on a weight-to-weight basis.

### 2.6. Particle Size by Dynamic Light Scattering

Average particle diameter for the formulations was determined by dynamic light scattering (DLS) using a Wyatt DynaPro 96-well plate reader (Santa Barbara, California). Formulations were dissolved in 0.9% saline at a concentration of 1 mg/mL, and 200 *μ*L of each sample was added to the wells of a 96-well plate. Average particle diameter was determined using 10 acquisitions per well with a 30-second acquisition time at 37°C for each sample.

### 2.7. Dialysis Stability Assays

Retention of the epothilone drug substance within the micelle formulation and the release profile of the drug substance over time, concentration, and pH were investigated using dialysis. Formulations were reconstituted at concentrations of 20 mg/mL and 0.2 mg/mL and placed in dialysis tubing with 3500 MWC pore size, which allows for retention of the micelles within the bag and release of the free drug from the bag into the reservoir. A volume of 3 mL of reconstituted formulation was placed into dialysis bags and then placed in a 300 mL reservoir containing 10 mM phosphate buffer and stirred for 6 hours at room temperature. HPLC assay for epothilone concentration of the formulation inside the bag was done before and after dialysis to determine the percentage of epothilone retained inside the dialysis bag over 6 hours. pH-dependent release was performed using 10 mM phosphate buffer at pH 3, 4, 5, 6, 7, 7.4, and 8 in the reservoirs during dialysis.

### 2.8. Epothilone D Rat Plasma Pharmacokinetics

The lead hydroxamic acid and cysteine containing formulations were compared to free epothilone D using a cannulated rat model by administering a 10 mg/kg dose by fast bolus to the jugular vein catheter. Female, Sprague-Dawley rats surgically modified with jugular vein catheters exteriorized posteriorly were purchased from Harlan Labs. Catheters were flushed and maintained with heparinized saline to ensure patency over the study period. The catheter port was used for both test article administration and blood collection. IT-147 pharmacokinetics at 20, 30, and 40 mg/kg were compared to epothilone D free drug at 20 mg/kg in the cannulated rat model. IT-147 was reconstituted in saline, while epothilone D free drug was reconstituted in cremaphor : ethanol : saline at 1 : 1 : 4 ratio. Dosing summaries for the rat pharmacokinetic studies are shown in Table 2. All in vivo studies were conducted under Institutional Guidelines. The Institutional Animal Care and Use Committee (IACUC), at the University of South Florida (USF), approved all rat in vivo study protocols. Animals were maintained and evaluated under pathogen-free conditions in accordance with USF College of Medicine IACUC standards of care. Approximately 300 *μ*L of blood was collected into K_2_EDTA tubes containing PMSF (final concentration 2.5 mM) at time points of 1, 5, 15, and 60 minutes after test article administration. Samples were centrifuged at 2000 RPM for 5 minutes to isolate plasma. Plasma was aliquoted into microcentrifuge tubes and frozen at −20°C until processed for HPLC/UV analysis. The following bioanalytical method is a modification of the procedure originally developed by Yuan et al. [30]. Bioanalytical sample preparation was performed by liquid : liquid extraction. 50 *μ*L of plasma sample was added to 600 *μ*L of* n*-butyl chloride and vortexed for 10 minutes. Samples were centrifuged at 10,000 RPM for 10 minutes and the organic supernatant was aliquoted into microcentrifuge tubes. The* n*-butyl chloride supernatant was evaporated to dryness using a turbovap with nitrogen flow at room temperature, and 75 *μ*L of a 40% acetonitrile in water solution was added to each tube. Samples were then vortexed for 5 minutes, aliquoted into HPLC vials, and processed using HPLC methodology as described in Section 2.3. Blank rat plasma containing PMSF was used to spike in epothilone D for standard curve preparation and processed as described for the plasma samples to determine concentration of epothilone D isolated from the samples collected from the cannulated rats. Noncompartmental analysis of the pharmacokinetic data was done using Phoenix WinNonlin version 6.3.

## 3. Results

### 3.1. Synthesis of Triblock Copolymers

All of the hydroxamate and cysteine triblock polymers used in this study were prepared by the ring-opening polymerization of *α*-amino acid* N*-carboxyanhydrides (NCAs) by a 12 kDa *α*-methoxy-*ω*-amine polyethylene glycol (mPEG12K-NH_2_) initiator followed by sequential functional group manipulations as shown in Figure 1. The quality of the triblock polymers is essential to the formulation of epothilone D. For this reason, the NCA monomers and hemitelechelic PEG were synthesized in-house. The NCAs were prepared by reacting the appropriate *α*-amino acid (side chain protected as necessary) with diphosgene in THF, filtration through celite, and precipitated from heptane. The PEG was prepared from the* N,N*-dibenzylethanolamine initiated polymerization of ethylene oxide followed by a methyl iodide quench. Heterogeneous hydrogenation with Pearlman's catalyst (Pd(OH)_2_/C) furnished the PEG as its free base. Polymerization of the protected cysteine or glutamate NCAs is performed first to install the stabilizing block; this free amine terminus serves as the initiator for the second NCA polymerization to equip the hydrophobic core block. The appropriate basic or acidic conditions are used to deprotect the amino acid side chains, while treatment of the D/L-glutamic acid *γ*-benzyl esters with hydroxylamine and LiOH facilitates the formation of the hydroxamic acid moiety (Figure 1). Aqueous GPC and ^1^H NMR of the final triblock polymers was used to confirm the desired degree of polymerization and purity.

### 3.2. Pilot Formulation and Physiochemical Characterization

**Figure 2:**
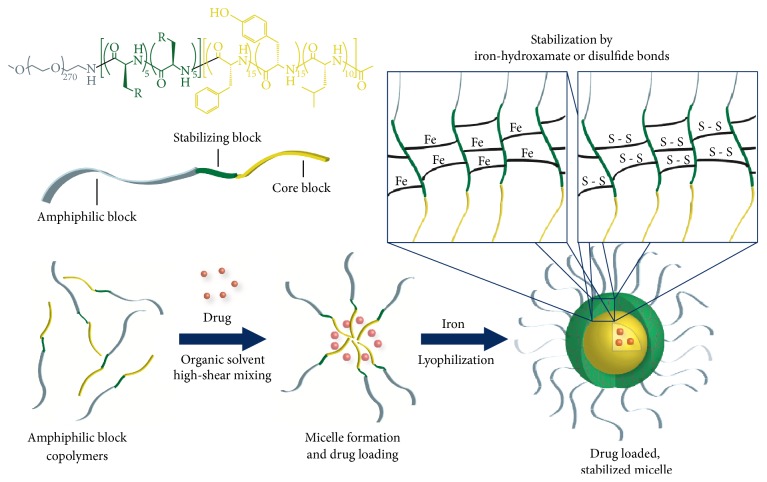
Formulation of epothilone D drug-loaded, stabilized micelles with triblock copolymers. The general formulation method includes formation of a polymer drug emulsion under high shear conditions, stabilization by iron-hydroxamate or disulfide bond formation, and lyophilization.

Epothilones B and D were selected for encapsulation using triblock copolymers. Initial analysis of pilot formulations demonstrated low drug loading and lack of stability of the epothilone B drug substance. Upon further investigation utilizing mass spectrometry, it was determined that the epoxide ring of epothilone B was unstable to all experimental formulation conditions. This was not completely surprising as stability of epothilone B both in vitro and in vivo has been addressed in the literature [31, 32]. Epothilone D, the alkene derivative of epothilone B (also known as desoxy-epothilone B), was selected as the preferred drug substance for encapsulation due to increased stability in aqueous environments and pH fluctuations. A small library of triblock copolymers was used to conduct pilot formulations of epothilone D drug-loaded micelles, with the ultimate goal to select IT-147, the lead for development of an epothilone D encapsulated polymer micelle formulation. Five of the polymers chosen for the library contained stabilizing blocks comprised of hydroxamic acid functionalized poly(glutamic acid), and five of the polymers contained poly(cysteine) amino acid moieties as stabilizing blocks (Figure 2, Table 1). All polymers contained a 12 kDa PEG and a mixture of hydrophobic amino acids with D and L isomers in the core block. Pilot formulations were characterized by determining formulation efficiency, epothilone D drug loading (% w/w), average particle diameter, and retention of epothilone D following dialysis above and below the critical micelle concentration (Table 1).

Epothilone D formulations containing polymer core blocks with a combination of phenylalanine, tyrosine, and leucine with hydroxamic acid (NHOH) stabilizing blocks demonstrated successful encapsulation of epothilone D based on greater than 80% retention of the API upon dialysis above the CMC, formulation efficiency above 50%, and average particle diameter between 70 and 100 nm. However, only the formulation utilizing a polymer with a core-forming block consisting of a mixture of phenylalanine/tyrosine/leucine (Table 1, entry number 1) demonstrated stability upon dilution when cross-linked with iron, which resulted in 60% retention of the drug after dialysis below the CMC. Epothilone D formulations containing polymer core blocks with aspartic acid resulted in low drug weight loading leading to inefficient formulations.

Polymers containing cysteine stabilizing blocks with leucine/tyrosine or phenylalanine/tyrosine core blocks demonstrated successful encapsulation of epothilone D with formulation efficiencies of 72% and 56%, average particle diameter of 68 and 86 nm, and API retention of 94% and 87% after dialysis above the CMC. The formulation with polymer core block D-Leu_20_/L-Tyr_20_ (this nomenclature represents a random copolymer comprised of 20 leucine and 20 tyrosine monomer units) demonstrated the greatest stability upon dilution for the cysteine-containing polymers with 38% retention when dialyzed below the CMC (Table 1, entry number 7). Cysteine-containing polymers with aspartic acid in the core did not result in the successful encapsulation of epothilone D. Based on the data presented in Table 1 formulations with NHOH-block-D-Phe_15_/L-Tyr_15_/D-Leu_10_ and Cysteine-block-D-Leu_20_/L-Tyr_20_ were chosen as the top candidates for lead selection of IT-147.

### 3.3. Selection of IT-147 Lead Formulation by Rat Pharmacokinetics

Stability of polymer micelles to dilution is one of the most important physiochemical characteristics for a formulation to be successful in vivo. Dialysis assays and other in vitro stability experiments provide important information and a cost effective means for screening formulations. However, the true measure of stability is the ability of the micelle to remain intact and circulate once injected into the bloodstream. It has been our experience that for screening purposes *C*_max_ and first hour of the distribution phase in plasma are highly predictive of the performance of the polymer micelle formulations. This allows for rapid assessment of the effectiveness of the encapsulation by comparing the micelle formulations to the free drug, while minimizing the total number of animals and samples needed for the study. This approach is beneficial for screening purposes for lead selection prior to full characterization of the pharmacokinetic properties of the lead formulation. The top two candidates from the pilot epothilone D formulations were evaluated, along with epothilone D free drug, using a cannulated rat model for pharmacokinetics. Selection of the lead epothilone D formulation, termed IT-147, was determined from the exposure of epothilone D to the plasma compartment by calculating the area under the concentration versus time curve (AUC) and maximum concentration (*C*_max_). To determine the elimination of the drug from the plasma compartment over the first hour, 10 mg/kg epothilone D was administered by fast bolus to the rat jugular vein catheter (Table 2). Plasma was isolated from blood collected at time points of 1, 5, 15, and 60 minutes and processed for epothilone D concentration by HPLC/UV, as shown in Figure 4. The dose of 10 mg/kg epothilone D equivalent was chosen to screen the formulations against the free drug due to toxicities that occur when dosing the free drug above 10 mg/kg, as noted in the toxicokinetic results described in Table 4. Epothilone D from the NHOH micelle formulation demonstrated a *C*_max_ of 42.7 *μ*g/mL at the 1 minute time point and an AUC_(0-1 h)_ of 18.9 h*∗μ*g/mL. Epothilone D from the Cys micelle formulation demonstrated a *C*_max_ of 34 *μ*g/mL at the 1-minute time point and an AUC_(0-1 h)_ of 10.6 h*∗μ*g/mL. Epothilone D free drug demonstrated a *C*_max_ of 19.7 *μ*g/mL at the 1 minute time point and an AUC_(0-1 h)_ of 5.2 h*∗μ*g/mL. The pharmacokinetics of epothilone D from the NHOH micelle formulation showed an increased *C*_max_ and AUC based on plasma circulation after 1 hour compared to the Cys formulation and epothilone D free drug in the distribution phase (Figure 4, Table 2). Based on this data the NHOH micelle was selected as IT-147, the lead formulation for scale up and development. The polymer used in IT-147, mPEG12K-NH-*b-p*-[D-Glu(NHOH)_5_-*co*-L-Glu(NHOH)_5_]-*b-p*-[D-Phe_15_-*co*-L-Tyr_15_-*co*-D-Leu_10_]-Ac, was termed ITP-103.

### 3.4. Scale-Up and Physiochemical Characterization of IT-147

With lead selection complete for the IT-147 formulation the next step was to scale the batch size from a pilot scale of 5 grams to a larger scale of 200 grams. This scale allowed for further physiochemical characterization, assessment of scalability for future larger scale batches, and further in vivo testing. Formulation of IT-147 on the 200 gram scale led to an 91% efficient process, with a drug loading of 4.4% on a 5% feed based on polymer weight. The average particle diameter was 75 nm as measured by DLS, displaying a narrow particle size distribution with *D*10/*D*50/*D*90 measurements of 29.7 nm, 34.5 nm, and 39.5 nm, respectively (Figure 5). Stability of the IT-147 formulation was tested by dialysis above the CMC and below the CMC and compared to the unstabilized NHOH formulation and epothilone D free drug (Figure 6). Dialysis of IT-147 above the CMC (20 mg/mL) demonstrated successful encapsulation with 90% of epothilone D remaining after dialysis, compared to 15% of epothilone D free dug retained after 6 hours of dialysis. Likewise, dialysis of IT-147 below the CMC (0.2 mg/mL) led to 60% retention of epothilone D, compared to 10% of epothilone D remaining from the unstabilized formulation. The release of epothilone D from IT-147 below the CMC based on pH-dependence was tested by dialysis in phosphate buffer between pH 3 and 8 over 6 hours (Figure 6). IT-147 demonstrated a pH-dependent release of epothilone D with 13, 15, 21, 39, 52, 64, and 66% remaining at pH 3, 4, 5, 6, 7, 7.4, and 8, respectively. Dialysis of the unstabilized NHOH formulation resulted in 10–15% epothilone D remaining over the pH ranging 3–8.

### 3.5. Pilot Toxicokinetics in a Cannulated Rat Model

A pilot dose range-finding toxicokinetic study in a cannulated rat model was performed to support future full scale toxicokinetic studies of IT-147. Doses of IT-147 at 20, 30 and 40 mg/kg were compared to epothilone D free drug at 20 mg/kg (Table 3, Figure 7) followed by monitoring of animal weight change, gross signs of clinical toxicity, and limited plasma sampling for pharmacokinetic analysis (Table 4). IT-147 was reconstituted in saline while epothilone D free drug was administered in a cremaphor : ethanol : saline mixture. Test articles were given by fast bolus to the rat jugular vein, and blood was collected at time points of 1 minute, 5 minutes, 15 minutes, 1 hour, 2 hours, 4 hours, and 6 hours. Epothilone D from IT-147 demonstrated a linear increase in maximum concentration and exposure to plasma compartment by area under the curve analysis (Table 4). IT-147 administered at 20 mg/kg epothilone D equivalent resulted in a *C*_max_ of 59.1 *μ*g/mL, an AUC of 53.5 h*∗μ*g/mL, a clearance rate of 373.7 mL/h/kg, and a terminal half-life of 4.3 hours. Animals were monitored for one week after test article administration and demonstrated no signs of gross clinical toxicity or significant weight fluctuation. In contrast, Epothilone D free drug administered at 20 mg/kg resulted in a *C*_max_ of 32.2 *μ*g/mL, an AUC of 8.3 h*∗μ*g/mL, a clearance rate of 2414.7 mL/h/kg, and a terminal half-life of 0.3 hours. Samples collected after the 1-hour time point were below the limit of quantitation for analysis. Animals were monitored for four days before test article administration and were euthanized on the fourth day due to welfare concerns. Animals demonstrated weight loss on average of 12% and clinical signs of dose limiting toxicities such as lethargy, impaired gait, orbital bleeding, porphyrin accumulation around the face, and disheveled fur. It was concluded that 20 mg/kg epothilone D free drug was above the maximum tolerated dose for these animals. IT-147 administered at 30 mg/kg epothilone D equivalent resulted in a *C*_max_ of 85.4 *μ*g/mL, an AUC of 82.9 h*∗μ*g/mL, a clearance rate of 361.8 mL/h/kg, and a terminal half-life of 5.1 hours. Animals were monitored for one week following test article administration. They demonstrated an average weight loss of 10% over the first four days, which started to recover between days five and seven. Animals in this dosing group demonstrated slight lethargy unless prompted to move about their cage. No orbital bleeding or gait impairment was noted for these animals. IT-147 administered at 40 mg/kg epothilone D equivalent resulted in a *C*_max_ of 107.6 *μ*g/mL, an AUC of 110.8 h*∗μ*g/mL, a clearance rate of 361.0 mL/h/kg, and a terminal half-life of 4.4 hours. Animals were monitored for four days after test article administration and were euthanized on the fourth day due to welfare concerns. Animals demonstrated weight loss on average of 14%, and clinical signs of dose limiting toxicities such as lethargy, impaired gait, orbital bleeding, porphyrin accumulation around the face, and disheveled fur. It was concluded that 40 mg/kg epothilone D from IT-147 was above the maximum tolerated dose for these animals. Taken together, this data demonstrated that the maximum tolerated dose for IT-147 was greater than 30 mg/kg but below 40 mg/kg. This data will be used to support full toxicokinetic studies with complete pharmacokinetic analysis in order to determine the NOAEL and maximum tolerated dose for IT-147.

## 4. Discussion

Enabling technologies for drug delivery have the potential to expand the utility of drug substances, especially cytotoxic chemotherapeutics. Polymer micelles have long been studied for these purposes; however they have demonstrated limited success in providing safer and more effective formulations. We hypothesized that parameters such as stability to dilution below the critical micelle concentration, particle size, and targeted drug release without the need for chemical conjugation or enzymatic activation are paramount to the success of micellar formulations. Encapsulation of epothilone D provided an opportunity to investigate stabilizing technologies for triblock copolymers using iron-hydroxamate or disulfide bonds in the stabilizing middle block. A small library of polymers that incorporate a mixture of D and L isomer amino acids in the core block for sequestration of epothilone D was used for pilot formulations. The D/L mix strategy is critical for forming a micelle core that is less rigid by breaking up the *α*-helix secondary structure.

Traditional polymer micelles inherently suffer from instability due to dilution and exposure to surfactant proteins and salt gradients when administered systemically. Instability leads to premature release of the drug substance that abrogates the benefits imparted by encapsulation in the micelle. To address the instability issue Intezyne has incorporated a stabilizing block between the amphiphilic PEG block and encapsulation core block. Two strategies were employed to investigate cross-linking stability for the micelle formulations. The first strategy was to functionalize a middle block by converting glutamic acid to hydroxamic acid (NHOH) moieties such that iron molecules form dative bonds between polymer strands effectively cross-linking the micelle and stabilizing the structure. The second strategy was to incorporate cysteine (Cys) amino acids in the middle block so that formulation could be performed in the presence of a reducing agent (DTT), and upon removal of the agent disulfide bonds could form between the cysteine molecules. Physiochemical parameters such as drug loading efficiency, particle size, and stability to dilution were used to screen the pilot formulations to determine the leads. Successful encapsulation was demonstrated with both NHOH and Cys polymers as determined by dialysis of the formulations when reconstituted above the critical micelle concentration (20 mg/mL). Polymers with leucine in the core block led to more efficient encapsulation of epothilone D, as well as the most stable formulations to dilution after cross-linking. When reconstituted at 0.2 mg/mL the lead formulations retained 60% (NHOH) and 38% (Cys) of the epothilone D inside the dialysis bag compared to less than 15% remaining for the same formulations without iron (NHOH) or DTT (Cys) present in the formulation. Ultimately, the lead NHOH polymer with the core of D-Phe_15_/L-Tyr_15_/D-Leu_10_, and the lead Cys polymer with the core L-Tyr_20_/D-Leu_20_ were selected for screening using a cannulated rat model for pharmacokinetics to determine the optimized lead for further characterization and development. The iron-hydroxamate stabilized formulation outperformed in the cannulated rat model and was chosen to scale up for further characterization.

The ability to scale up production of materials and drug product is critical to the development of nanoparticle formulations. Preclinical animal models and studies necessary for enabling Investigational New Drug (IND) applications can require hundreds of grams to kilograms of material. Therefore, we demonstrated production of IT-147 drug product on the 200 gram scale for complete physiochemical characterization and preliminary dose range-finding toxicokinetic studies, with future studies focused on determining antitumor efficacy and toxicokinetics with full pharmacokinetics in dose range-finding studies. When produced on scale the formulation efficiency increased to over 90 percent, likely due to a decrease in loss during transfer, tangential flow filtration, and sterile filtration. Particle size analysis demonstrated an average hydrodynamic diameter of 75 nm, with a narrow particle size distribution. Particle size has been shown to play a key role in influencing nanoparticle circulation time, accumulation in the tumor environment by the enhanced permeability and retention (EPR) effect, depth of penetration into the tumor, and cellular entry mechanism. This was taken into account when tuning the block lengths of the polymer monomers such that the self-assembly of the micelle around the drug substance would yield a nanoparticle with optimal size characteristics.

**Figure 3:**
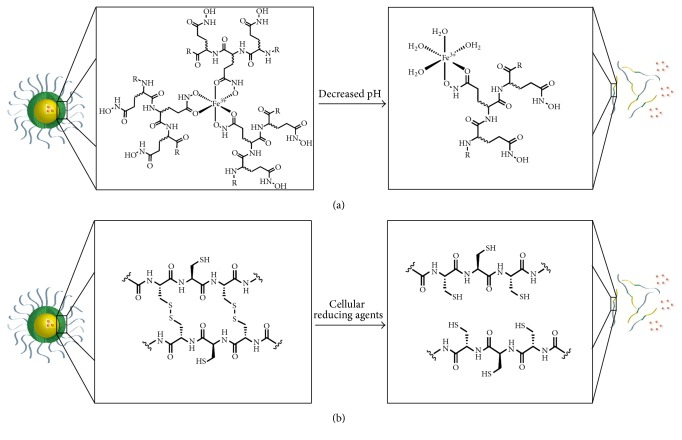
Mechanism of drug payload release from the polymer micelle formulations. (a) Stabilization by iron-hydroxamate bonds between polymer chains is reversed by a decrease in pH. (b) Stabilization by disulfide bonds between polymer chains is reversed by intracellular reducing agents.

**Figure 4:**
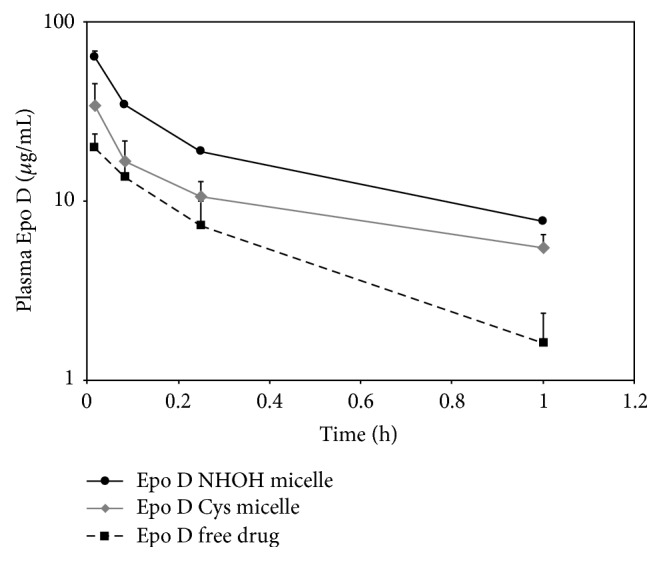
Plasma pharmacokinetics of epothilone D from lead pilot formulations and free drug doses at 10 mg/kg. Screening of the first hour of plasma circulation demonstrates increased *C*_max_ and AUC for the NHOH micelle formulation.

**Figure 5:**
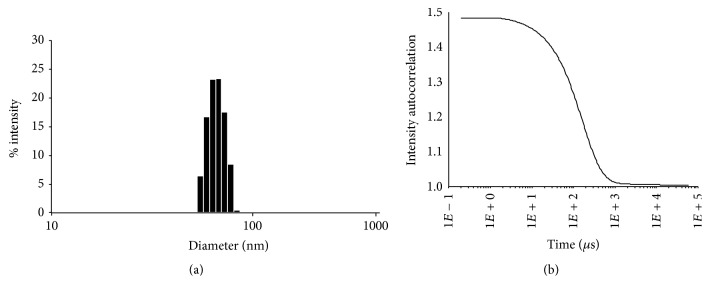
Particle size analysis of IT-147 by dynamic light scattering. Particle size distribution histogram (a) and correlation function (b) for particle size analysis demonstrates a monomodal particle size distribution at 75 nm in diameter.

**Figure 6:**
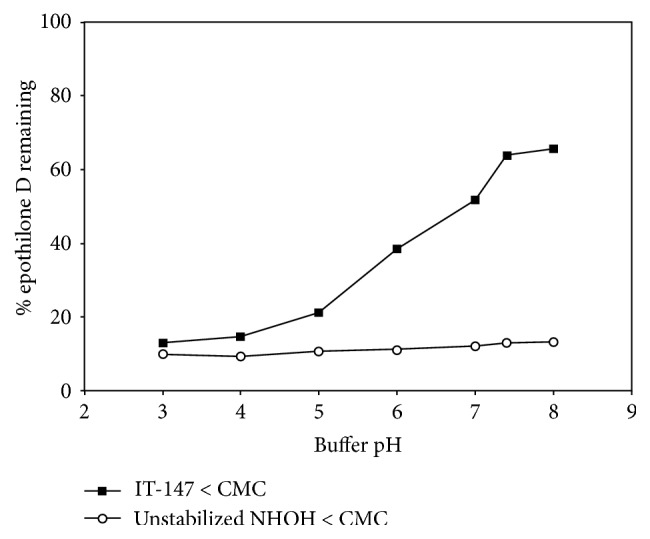
pH-dependent release of IT-147 by dialysis. The iron-stabilized IT-147 formulation demonstrates stability at physiological pH and a pH-dependent release of epothilone D as the pH decreases, whereas the unstabilized formulation releases the drug payload at all pH levels tested within the 6-hour dialysis assay.

**Figure 7:**
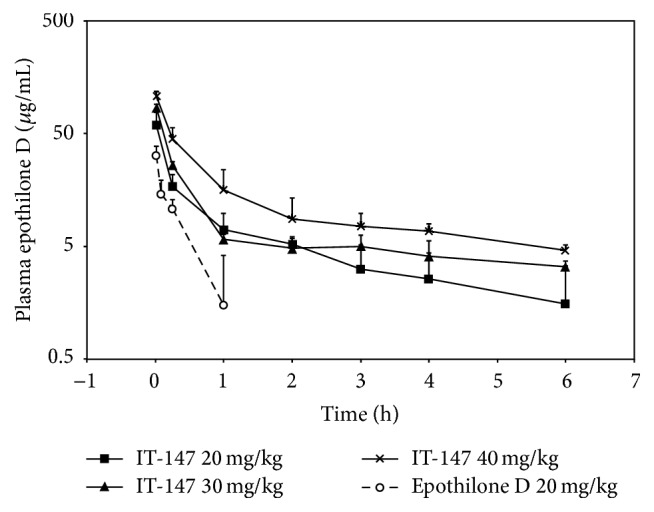
Plasma pharmacokinetics from the pilot toxicokinetic dose range-finding screen of IT-147. Plasma samples were isolated and assayed for epothilone D content during the pilot toxicokinetic screen to support full toxicokinetic and pharmacokinetic studies. IT-147 demonstrated superior pharmacokinetics compared to free drug at 20 mg/kg, and a linear increase in *C*_max_ and AUC in dosing from 20 to 40 mg/kg.

In addition to the size and stability of the nanoparticle formulation, the release of the drug substance is critical to providing an efficacious drug product. A distinct advantage of using iron to chelate hydroxamic acid moieties for micelle stability is the reversibility of this interaction based on pH, as depicted graphically in Figure 3. A pH-dependent targeted release of drug substance payload for oncology applications is hypothesized to lead to increased efficacy with a greater exposure to the tumor compartment, while keeping the drug exposure low to other critical compartments should lead to decreased toxicity. In vitro we have demonstrated the pH-dependent release of epothilone D from the micelle using dialysis in buffers with a range of pH from 3 to 8. IT-147 formulation was reconstituted at 0.2 mg/mL and dialyzed for 6 hours in the corresponding buffers. This release profile serves as proof of concept for targeted release. It has been well documented that the pH of most tumor microenvironments is in the range of 5.5 to 7 [25, 33–37]. In addition, cellular uptake by endocytosis can lead to the formation of lysosomes, which can have an internal pH of 4 to 5 [26]. It is of great interest to determine the precise location and mechanism for release of the drug payload from the micelle, whether it is intracellular or extracellular. However, this is a highly complex question that despite ongoing experimentation has not been adequately addressed at this time.

An important mechanism for nanoparticles to accumulate in the tumor compartment is prolonged circulation in the blood/plasma compartment. A dose-dependent pharmacokinetic study in cannulated rats was done to assess the plasma pharmacokinetics of epothilone D from IT-147. A dose of 20 mg/kg epothilone D from IT-147 was well tolerated with no observed adverse events and resulted in greater than 6-fold increase in plasma exposure compared to free drug. Administration of epothilone D free drug at 20 mg/kg was determined to be above the maximum tolerated dose in these animals and resulted in weight loss greater than 10%, with clinical signs of toxicity such as lethargy, gait impairment, and retro-orbital bleeding. Pharmacokinetic analysis of the dose dependency from IT-147 showed near linearity for AUC and *C*_max_, with increased half-life and decreased clearance compared to epothilone D free drug. The extended circulation in the plasma compartment can be directly attributed to the increased stability of the micelle from iron-mediated cross-linking. In addition to imparting stability, the geometrical arrangement of iron atoms around the core of the micelle also provides superparamagnetic properties that allow for positive contrast using T_1_-weighted magnetic resonance imaging. This theranostic strategy could provide a potential imaging biomarker for clinical applications.

Taken together, formulation of IT-147 presents a significant improvement in delivery of epothilone D and is positioned for development for oncology applications. Future preclinical studies will focus on determining the full toxicokinetic profile, antitumor efficacy, and tissue biodistribution of epothilone D delivered by IT-147.

## Figures and Tables

**Table 1:** Physiochemical characterization of epothilone D pilot formulations with iron-hydroxamate or disulfide bond stabilization. Formulations were performed with each polymer listed as an entry number and characterized by drug weight loading, efficiency, particle size, and dialysis above and below the critical micelle concentration. Entry numbers 1 and 7 represent the leads selected for screening, and entry number 2 represents the scaled-up lead formulation.

Entry number	Polymer core block	Polymer stabilizing block	Weight loading (% w/w)	Efficiency (%)	Average diameter (nm)	Dialysis > CMC (% remaining)	Dialysis < CMC (% remaining)
**1**	D-Phe_15_/ L-Tyr_15_/ D-Leu_10_	NHOH	3.4	66	70	90	60
**2**	D-Phe_15_/ L-Tyr_15_/ D-Leu_10_	NHOH	4.4	91	75	94	68
3	D-Phe_15_/ L-Tyr_25_	NHOH	3.3	64	70	80	25
4	D-Phe_15_/ L-Tyr_20_/ D-Leu_5_	NHOH	3.0	58	91	91	28
5	D-Phe_15_/ L-Tyr_20_/ D-Asp_5_	NHOH	1.7	33	97	82	28
6	D-Phe_10_/ L-Tyr_20_/ D-Asp_10_	NHOH	0.9	17	>200	55	<10
**7**	L-Tyr_20_/ D-Leu_20_	Cysteine	3.7	72	68	94	38
8	D-Phe_15_/ L-Tyr_25_	Cysteine	2.9	56	86	87	11
9	D-Phe_15_/ L-Asp_25_	Cysteine	0.1	<1	35	ND	ND
10	L-Tyr_20_/ D-Asp_20_	Cysteine	0.1	<1	>200	47	<10
11	D-Leu_15_/ L-Tyr_20_/ D-Asp_20_	Cysteine	0.5	1	>200	ND	ND

**Table 2:** Plasma pharmacokinetic screening of pilot epothilone D formulations compared to free drug. A cannulated rat model was used to determine the AUC, *C*_max_, and Clast of epothilone D in plasma for the first hour after administration.

Test article	Animals per group	Dose (mg/kg)	AUC_(0-1 h)_ (h*∗µ*g/mL)	*C*_max_ (*µ*g/mL)	Clast (*µ*g/mL)
NHOH micelle	3	10	18.9	42.7 ± 4.9	7.7 ± 0.2
Cys micelle	3	10	10.6	34.0 ± 11.0	4.8 ± 1.1
Free drug	3	10	5.2	19.7 ± 4.1	1.6 ± 0.8

**Table 3:** Dosing summary for rat pharmacokinetic studies. Epothilone D free drug was dosed at 10 and 20 mg/kg. IT-147 was dosed at 10, 20, 30, and 40 mg/kg epothilone D equivalent or 500, 1000, 1500, and 2000 mg/kg formulation, respectively.

Test article	Dose epothilone D (mg/kg)	Dose IT-147 (mg/kg)	Epothilone D concentration (mg/mL)	IT-147 concentration (mg/mL)	Test article volume (mL)
Epo D	10	0	1.15	0	2
Epo D	20	0	2.3	0	2
IT-147	10	500	1.15	57.5	2
IT-147	20	1000	2.30	115	2
IT-147	30	1500	3.45	173	2
IT-147	40	2000	4.60	230	2

**Table 4:** Pilot toxicokinetic dose range-finding study for IT-147 formulation. IT-147 was administered at 20, 30, and 40 mg/kg epothilone D equivalent compared to free drug at 20 mg/kg. Limited pharmacokinetic sampling was used to determine *C*_max_, AUC, clearance, and half-life data for each animal up to 6 hours after test article administration.

Test article	Dose (mg/kg)	Animals per group	*C*_max_ (*µ*g/mL)	AUC_(0–6 h)_ (h*∗µ*g/mL)	Clearance (mL/h/kg)	Half-life (h)	Dose limiting Toxicities
Epo D	20	6	32.2	8.3	2414.7	0.3	>10% w.l. lethargy impaired gait orbital-bleeding
IT-147	20	4	59.1	53.5	373.7	4.3	None
IT-147	30	4	85.4	82.9	361.8	5.1	10% w.l. slight-lethargy
IT-147	40	4	107.6	110.8	361.0	4.4	>10% w.l. lethargy impaired gait orbital-bleeding

## References

[1] Reichenbach H., Höfle G. (1993). Biologically active secondary metabolites from myxobacteria. *Biotechnology Advances*.

[2] Hofle G. (1996). Epothilone A and B—novel 16-membered macrolides with cytotoxic activity: isolation, crystal structure, and conformation in solution. *Angewandte Chemie*.

[3] Gerth K., Bedorf N., Höfle G., Irschik H., Reichenbach H. (1996). Epothilons A and B: antifungal and cytotoxic compounds from Sorangium cellulosum (Myxobacteria) production, physico-chemical and biological properties. *The Journal of Antibiotics*.

[4] Bollag D. M., McQueney P. A., Zhu J. (1995). Epothilones, a new class of microtubule-stabilizing agents with a taxol- like mechanism of action. *Cancer Research*.

[5] Kowalski R. J., Giannakakou P., Hamel E. (1997). Activities of the microtubule-stabilizing agents epothilones A and B with purified tubulin and in cells resistant to paclitaxel (Taxol®). *The Journal of Biological Chemistry*.

[6] Nicolaou K. C., Winssinger N., Pastor J. (1997). Synthesis of epothilones A and B in solid and solution phase. *Nature*.

[7] Lee F. Y. F., Borzilleri R., Fairchild C. R. (2001). BMS-247550: a novel epothilone analog with a mode of action similar to paclitaxel but possessing superior antitumor efficacy. *Clinical Cancer Research*.

[8] Chou T.-C., Dong H., Rivkin A. (2003). Design and total synthesis of a superior family of epothilone analogues, which eliminate xenograft tumors to a nonrelapsable state. *Angewandte Chemie*.

[9] Goodin S., Kane M. P., Rubin E. H. (2004). Epothilones: mechanism of action and biologic activity. *Journal of Clinical Oncology*.

[10] O'Reilly T., McSheehy P. M. J., Wenger F. (2005). Patupilone (epothilone B, EPO906) inhibits growth and metastasis of experimental prostate tumors in vivo. *Prostate*.

[11] Fumoleau P., Coudert B., Isambert N., Ferrant E. (2007). Novel tubulin-targeting agents: anticancer activity and pharmacologic profile of epothilones and related analogues. *Annals of Oncology*.

[12] Su D.-S., Balog A., Meng D. (1997). Structure-activity relationship of the epothilones and the first in vivo comparison with paclitaxel. *Angewandte Chemie*.

[13] Chou T.-C., Zhang X.-G., Balog A. (1998). Desoxyepothilone B: an efficacious microtubule-targeted antitumor agent with a promising in vivo profile relative to epothilone B. *Proceedings of the National Academy of Sciences of the United States of America*.

[14] Chou T.-C., Zhang X.-G., Harris C. R. (1998). Desoxyepothilone B is curative against human tumor xenografts that are refractory to paclitaxel. *Proceedings of the National Academy of Sciences of the United States of America*.

[15] Blum W., Aichholz R., Ramstein P. (2001). In vivo metabolism of epothilone B in tumor-bearing nude mice: identification of three new epothilone B metabolites by capillary high-pressure liquid chromatography/mass spectrometry/tandem mass spectrometry. *Rapid Communications in Mass Spectrometry*.

[16] Chou T.-C., O'Connor O. A., Tong W. P. (2001). The synthesis, discovery, and development of a highly promising class of microtubule stabilization agents: curative effects of desoxyepothilones B and F against human tumor xenografts in nude mice. *Proceedings of the National Academy of Sciences of the United States of America*.

[17] Larkin J. M. G., Kaye S. B. (2007). Potential clinical applications of epothilones: a review of phase II studies. *Annals of Oncology*.

[18] Overmoyer S. W. B., Kaufman P. A., Doyle T. (2005). Phase II trial of KOS-862 (epothilone D) in anthracycline and taxane pretreated metastatic breast cancer. *Journal of Clinical Oncology*.

[19] Yee T. L. L., Villalona-Calero M., Rizvi N. (2005). A phase II study of KOS-862 (epothilone D) as second-line therapy in non-small cell lung cancer. *Journal of Clinical Oncology*.

[20] Feldman D. R., Kondagunta G. V., Ginsberg M. S. (2007). Phase II Trial of ixabepilone in patients with cisplatin-refractory germ cell tumors. *Investigational New Drugs*.

[21] McMeekin S., Dizon D., Barter J. (2015). Phase III randomized trial of second-line ixabepilone versus paclitaxel or doxorubicin in women with advanced endometrial cancer. *Gynecologic Oncology*.

[22] Rugo H. S., Barry W. T., Moreno-Aspitia A. (2015). Randomized phase III trial of paclitaxel once per week compared with nanoparticle albumin-bound nab-paclitaxel once per week or ixabepilone with bevacizumab as first-line chemotherapy for locally recurrent or metastatic breast cancer: CALGB 40502/NCCTG N063H (Alliance). *Journal of Clinical Oncology*.

[23] Venditto V. J., Szoka F. C. (2013). Cancer nanomedicines: so many papers and so few drugs!. *Advanced Drug Delivery Reviews*.

[24] Stylianopoulos T., Jain R. K. (2015). Design considerations for nanotherapeutics in oncology. *Nanomedicine: Nanotechnology, Biology, and Medicine*.

[25] Zhang X., Lin Y., Gillies R. J. (2010). Tumor pH and its measurement. *Journal of Nuclear Medicine*.

[26] Mindell J. A. (2012). Lysosomal acidification mechanisms. *Annual Review of Physiology*.

[27] Wang J., Li S., Luo T., Wang C., Zhao J. (2012). Disulfide linkage: a potent strategy in tumor-targeting drug discovery. *Current Medicinal Chemistry*.

[28] (K.T. Sill, FL, US), Vojkovsky, Tomas (Palm Beach Gardens, FL, US), Carie, Adam (Ruskin, FL, US), Block copolymers for stable micelles, in, Intezyne Technologies, Inc. (Tampa, FL, US), United States, 2015

[29] Costich T. L. C., Semple A., Sullivan J. E. (2016). IT-143, a polymer micelle nanoparticle, widens therapeutic window of daunorubicin. *Pharmaceutical Nanotechnology*.

[30] Yuan L., Fu Y., Zhang D. (2014). Use of a carboxylesterase inhibitor of phenylmethanesulfonyl fluoride to stabilize epothilone D in rat plasma for a validated UHPLC-MS/MS assay. *Journal of Chromatography B: Analytical Technologies in the Biomedical and Life Sciences*.

[31] Nicolaou K. C., Sasmal P. K., Rassias G. (2003). Design, synthesis, and biological properties of highly potent epothilone B analogues. *Angewandte Chemie*.

[32] Altman G. H. K.-H., Muller R., Mulzer J., Prantz K. (2009). *The Epothilones: An Outstanding Family of Anti-Tumor Agents*.

[33] Tannock I. F., Rotin D. (1989). Acid pH in tumors and its potential for therapeutic exploitation. *Cancer Research*.

[34] Leeper D. B., Engin K., Thistlethwaite A. J. (1994). Human tumor extracellular pH as a function of blood glucose concentration. *International Journal of Radiation Oncology, Biology, Physics*.

[35] Engin K., Leeper D. B., Cater J. R., Thistlethwaite A. J., Tupchong L., Mcfarlane J. D. (1995). Extracellular pH distribution in human tumours. *International Journal of Hyperthermia*.

[36] Ojugo A. S. E., McSheehy P. M. J., McIntyre D. J. O. (1999). Measurement of the extracellular pH of solid tumours in mice by magnetic resonance spectroscopy: a comparison of exogenous 19F and 31P probes. *NMR in Biomedicine*.

[37] van Sluis R., Bhujwalla Z. M., Raghunand N. (1999). In vivo imaging of extracellular pH using 1H MRSI. *Magnetic Resonance in Medicine*.

